# Field-Tunable 0-π-Transitions in SnTe Topological Crystalline Insulator SQUIDs

**DOI:** 10.1038/s41598-018-38008-1

**Published:** 2019-02-13

**Authors:** Joachim Schönle, Kiril Borisov, Robin Klett, Denis Dyck, Franck Balestro, Günter Reiss, Wolfgang Wernsdorfer

**Affiliations:** 1 Institut Néel, CNRS and University Grenoble-Alpes, 25 Rue des Martyrs, F-38042 Grenoble, France; 2 Center for Spinelectronic Materials & Devices, Physics Department, Bielefeld University, Universitätsstraße 25, D-33615 Bielefeld, Germany; 3 Physikalisches Institut (PHI), Karlsruhe Institute of Technology (KIT), Wolfgang-Gaede-Straße 1, D-76131 Karlsruhe, Germany; 4 Institute of Nanotechnology (INT), Karlsruhe Institute of Technology (KIT), Hermann-von-Helmholtz-Platz 1, D-76334 Eggenstein-Leopoldshafen, Germany

## Abstract

The manifestation of spin-orbit interactions, long known to dramatically affect the band structure of heavy-element compounds, governs the physics in the surging class of topological matter. A particular example is found in the new family of topological crystalline insulators. In this systems transport occurs at the surfaces and spin-momentum locking yields crystal-symmetry protected spin-polarized transport. We investigated the current-phase relation of SnTe thin films connected to superconducting electrodes to form SQUID devices. Our results demonstrate that an assisting in-plane magnetic field component can induce 0-π-transitions. We attribute these findings to giant g-factors and large spin-orbit coupling of SnTe topological crystalline insulator, which provides a new platform for investigation of the interplay between spin-orbit physics and topological transport.

## Introduction

Topological states of matter are researched in a large variety, ranging from 1D nanowire systems with strong spin-orbit coupling^1,2^ over 2D quantum spin Hall insulators^3,4^ to 3D topological insulators^5–7^ as the most common examples of this quickly emerging field. Topological crystalline insulators (TCI)^8^, with SnTe as a representative model material, constitute a new class of 3D materials within this widespread family, for which the topological properties are governed by mirror symmetries of the crystal lattice rather than time-reversal symmetry, giving rise to multiple Dirac surface channels with spin-momentum locking^9–11^. A fundamental and common interest in these states of matter is based upon the interplay of spin-polarized surface channels and superconducting pairing, ever since the possible realization of formerly elusive topological superconductivity in hybrid systems of such materials and common s-wave superconductors was predicted^12^. One of the particularly enticing prospects of this is the conceptual implementation of topological quantum computing^13^, which is enabled by non-Abelian and delocalized quasiparticle excitations commonly referred to as Majorana zero modes^14^. The scope of possible effects in such structure, related to unconventional pairing and phase relations, has been recently extended because of a more complete picture of the role of spin-orbit coupling in low-dimensional electron systems, most notable the similarities between Rashba-type spin splitting^15^ and topological spin-momentum locking. In this context, the impact of Zeeman fields has been used particularly as a driving force between trivial and unconventional regimes in theoretical proposals^16–18^ as well as experimental demonstrations^19–21^. This approach is fuelled by the experimental consent that the contributions of topological surface states are difficult to isolate from non-depleted bulk channels in common transport measurements. The latter are hence often dominated by trivial bulk characteristics^22–24^. Accordingly, both the realization of finite-momentum Cooper pairing^19,21^ and more demonstratively the occurrence of fractional^20,25^ and half-flux^19,20^ offset of Josephson junctions have been reported under applied in-plane magnetic field in related systems.

It is well established that the current-phase relationship (CPR) of a superconductor-insulator-superconductor (SIS) junction is sinusoidal in nature, following $\,{I}_{{\rm{J}}}({\rm{\phi }})={I}_{{\rm{c}}}\,\sin ({\rm{\phi }})$, which expresses itself as vanishing supercurrent and a non-degenerate minimum of the Josephson energy at ${\rm{\phi }}=0$. The sinusoidal shape is usually not preserved in spatially extended superconductor-normal metal-superconductor (SNS) junction, which allows for the evaluation of the transport channels by means of characteristic line shapes^26^, but the ${\rm{\phi }}=0$ behaviour is a stable mechanism in conventional systems. A Josephson junction with a Josephson energy offset of a fractional flux quantum is known as a φ_0_-junction and a Josephson junction with a Josephson energy offset of exactly half-integer flux quantum is known as a π–junction. Deviations in form of φ_0_-junctions and/or 0-π-transitions have been observed in devices of InSb nanowire quantum dots^25^, carbon nanotube quantum dots^27^, ferromagnetic layers^28^, spin valves^29^ and superconducting materials with different gap symmetry (*s*_±_ iron picnitides^30^ and d-wave cuprates^31^).

We build upon our previous experimental work on SQUIDs made of two thin film (001)-textured SnTe TCI Josephson junctions coupled to superconducting electrodes^24^. In this paper, we present our measurements of the CPR of these devices under applied in-plane magnetic fields.

We observe that the phase of the Josephson current can be shifted away from zero by an assisting in-plane-magnetic field and undergo continuous and field-controllable phase transitions of π. These findings are discussed in the context of order parameter oscillations in the TCI-based weak links. Applied magnetic fields have significant impact on materials with strong spin-orbit interaction and can induce finite Cooper pair momentum. The tuning of the latter then causes phase shifts of each Josephson junction^17–19,21,32–34^.

## Results

**Figure 1:**
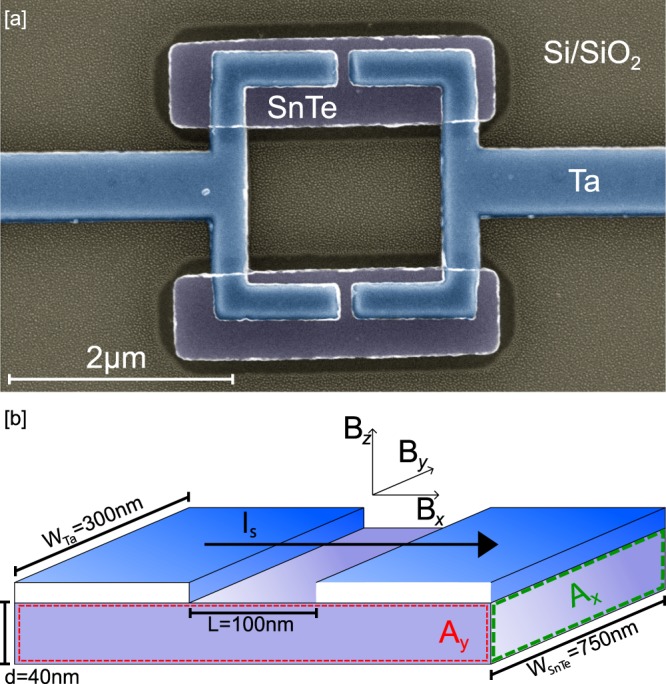
The SQUID device with SnTe weak links. (**a**) False-color SEM image of the measured SQUID device of ≈ 4 μm^2^ loop area. Josephson junctions are approximately 100 nm long and formed by 40 nm thick SnTe film. (**b**) Schematic of one of the Josephson junctions forming the SQUID, corresponding to the dashed black box in [a]. The alignment within the magnetic field is presented.

The measured device consists of a 40 nm layer of SnTe and 30 nm Ta electrodes on top, shaped as a SQUID ring of ≈ 4 μm^2^ loop area with 300 nm arms and 100 nm gaps forming the junctions as shown in Fig. 1a. A schematic of the device in the coordinate system of the magnetic field is shown in Fig. 1b.

**Figure 2:**
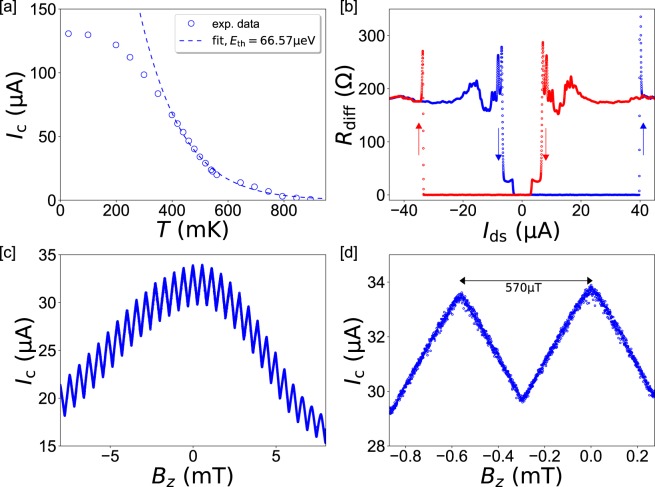
Critical current and perpendicular-field response of the device. (**a**) *I*_c_(*T*) measurement and the analytic fit described in the main text yield a Thouless energy of ${E}_{{\rm{th}}}\approx 67\,{\rm{\mu }}\text{eV}$. (**b**) Trace-retrace *R*(*I*) measurement at $T\cong 500\,{\rm{mK}}$. Thermal hysteresis is still significant due to the large *I*_c_, but limited compared to lower temperatures. The normal resistance here is dominated by simultaneous switching of the leads, while the weak links have ${R}_{{\rm{n}}}\approx 5\,{\rm{\Omega }}$. (**c**) *I*_c_(*B*_*z*_) SQUID modulation persists in a broad field range, here measured at *T* = 500 mK. (**d**) Close-up of the data in (**c**). Small modulation depth and a triangular CPR is observed, which is attributed to large kinetic inductance in the hybrid thin film device and explained in the main text.

The temperature dependence of the critical current is measured (Fig. 2a) and fitted with the law with ${I}_{{\rm{c}}}(T)\propto \sqrt{T}{e}^{-\frac{2\pi {k}_{{\rm{B}}}T}{{E}_{{\rm{th}}}}}$^35–37^. The latter describes the limit of long SNS junctions with $L > {\xi }_{{\rm{N}}}$ in an elevated temperature regime ${k}_{{\rm{B}}}T\ge {E}_{{\rm{th}}}/2{\rm{\pi }}$ and results in a fairly good fit for temperatures $T\ge 400\,{\rm{mK}}$. The Thouless energy ${E}_{{\rm{th}}}\approx 67\,{\rm{\mu }}\text{eV}$ is deduced from the fit, which represents the smaller and thus dominant energy scale of the junction, as the superconducting gap is given by ${\Delta }=1.76\,{k}_{{\rm{B}}}{T}_{{\rm{c}}}^{{\rm{Ta}}}\approx 380\,{\rm{\mu }}\mathrm{eV}$ for a transition temperature ${T}_{{\rm{c}}}^{{\rm{Ta}}}\approx 2.5\,{\rm{K}}$ of the superconducting Ta film. The superconducting coherence length can be estimated as ${\xi }_{{\rm{N}}}=L\sqrt{\frac{{E}_{{\rm{th}}}}{{\rm{\Delta }}}}\approx 43\,{\rm{nm}}$^38^, which places our device in the moderately long junction regime $L > {\xi }_{{\rm{N}}}$. The coherence length increases slightly with increasing temperature and places the junction towards the intermediate regime in qualitative agreement with literature^26,37,39^. With the low-temperature limit for infinitely long junctions^40^ $e{R}_{{\rm{n}}}{I}_{{\rm{c}}}\ge 10.82\,{E}_{{\rm{th}}}$ one can derive ${E}_{{\rm{th}}}\approx 60\,{\rm{\mu }}\mathrm{eV}$ which confirms the obtained Thouless energy from the fit above. The strong proximity-induced superconductivity of the SnTe/Ta gives rise to a large critical current ${I}_{{\rm{c}}}\approx 130\,{\rm{\mu }}A$ at the base temperature $T=30\,{\rm{mK}}$, which exceeds significantly the constant, cooling-driven retrapping current *I*_r_. The switching behaviour is shown in a ${R}_{{\rm{diff}}}({\rm{I}})$ plot for $T\lesssim 500\,{\rm{mK}}$ (Fig. 2b). Tantalum is chosen as a superconducting electrode in our structure because it provides strong induced superconductivity and high critical currents through the SnTe weak-link. High in-plane critical fields are necessary to induce 0-π transitions. The strong spin-orbit coupling of Ta does not influence the observed effects because the switching characteristic is determined by the SnTe weak-link. The latter is justified by the difference between the critical temperature of SnTe (*T*_C_^SnTe^ ≈ 900 mK) and the critical temperature of Ta (*T*_C_^Ta^ ≈ 2.5 K).

The thermal avalanche from the switching at low temperatures causes transitions of the nearby superconducting structures into a resistive state as well which leads to resistance values not solely containing the weak link. In order to limit the disturbing influence of large thermal hysteresis effects, further measurements are performed at elevated temperatures $T\ge 500\,{\rm{mK}}$. From measurements close to ${T}_{{\rm{c}}}^{{\rm{SnTe}}}\approx 900\,{\rm{mK}}$, we deduce normal state resistance ${R}_{{\rm{n}}}\approx 5\,{\rm{\Omega }}$ of the weak links used in the relation above, which corresponds well to supporting resistivity measurements of an SnTe Hall bar structure (see Supplementary Information). The extracted normal state resistance of our devices demonstrates multichannel transport. Therefore, there is contribution from the bulk states of the TCI composition as well similar to other reports^41,42^. Our previous investigations on the bare SnTe devices have shown the manifestation of the TCI surface states through weak-antilocalization measurements^24^.

Subsequently, the CPR response to magnetic field is probed. Application of an out-of-plane magnetic field *B*_*z*_ generates a SQUID modulation whose periodicity corresponds well to the spatial dimensions of the device for purely 2π-dominated transport (Fig. 2b). The absence of any 4π-periodic contributions in DC measurements of topological matter is a commonly reported effect, which results from bulk-shunting and therefore poisoning of surface states^22–24^ on long measurement time scales. A less-dissipative approach is provided by RF measurements, where the 4π-effect expresses itself as the vanishing of odd-integer Shapiro steps, as recently demonstrated for strained 3D topological insulator HgTe^43^, HgTe quantum wells^44^ and Dirac semimetal Bi_1−*x*_Sb_*x*_^42^.

The CPR shows strongly reduced SQUID modulation depth (Fig. 2b). Most trivially, such behaviour can stem from asymmetric junctions with ${I}_{{\rm{c}}1}\ne {I}_{{\rm{c}}2}$, but our fabrication scheme should yield reasonably symmetric devices, for which asymmetry arises only microscopically. Here, the effect is therefore attributed to strong kinetic effects in the SnTe/Ta hybrid, which also explains the triangular shape of the CPR^45^. Kinetic effects can occur in superconducting junctions with ${\rm{L}}\ge {\xi }_{{\rm{N}}}$, which applies in our devices. From the slope ${\rm{d}}{{\Phi }}_{{\rm{SQ}}}/{I}_{{\rm{c}}}$ we derive the inductance of ${L}_{{\rm{k}}}\approx 450\,{\rm{pH}}$ and a corresponding screening factor ${\beta }_{{\rm{k}}}=\frac{2\pi {L}_{{\rm{k}}}{I}_{{\rm{c}}}}{{{\Phi }}_{0}}\,\approx 20\gg 1$, which dominates over geometric influences ${\beta }_{{\rm{geom}}}\approx 0.17$. Here, the large *β*_k_ arises as consequence of both the strong proximity-coupling with large critical currents and the large *L*_k_ in our devices. There is, however, no reason to assume the 0-π-effect originates from the large kinetic inductance and the Ta, which constitutes a common (type-1) s-wave superconductor. As the critical temperature of the SnTe weak links is approached, the critical current and hence *β*_k_ decreases. Indeed, we observe that close to ${T}_{{\rm{c}}}^{{\rm{SnTe}}}\approx 900\,{\rm{mK}}$ the CPR shows the classical cosine-like flux dependence of the SQUID. More details on the CPR and *R*_n_ can be found in the Supplementary Information.

**Figure 3:**
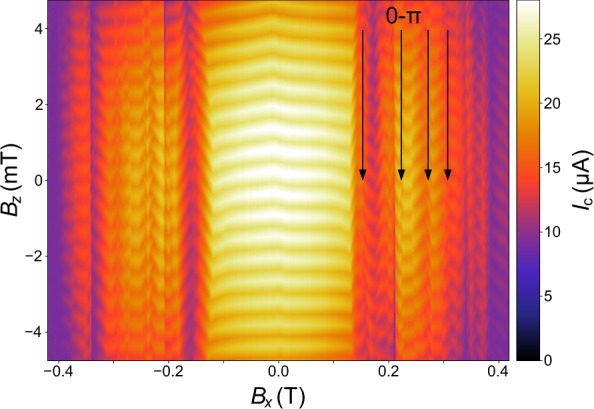
Modulation of the SQUID oscillation in additional in-plane magnetic fields. *I*_c_(*B*_*x*_, *B*_*z*_) SQUID modulation with additional in-plane field *B*_*x*_ at *T* = 500 mK. The linear drift of the pattern due to sample alignment was corrected by rotation. For in-plane fields $|{B}_{x}|\le 155\,{\rm{mT}}$, a non-linear shift of the CPR is observed, attributed to microscopic asymmetry. For $|{B}_{x}|\ge 155\,{\rm{mT}}$, a transitional regime is reached, where continuous field-induced 0-π-transitions occur, whose positions are indicated with black arrows.

Significant altering of the conventional modulation pattern is observed when the device is subject to an additional in-plane magnetic field *B*_*x*_ (see Fig. 3). A linear drift in the (*B*_*x*_, *B*_*z*_)-plane is corrected (here and for all following images containing in-plane fields) by rotation, as this constitutes the impact of non-perfect sample alignment within the magnetic field axes, which is confirmed by measurements in different field directions and a repeated measurement during a second cool down.

For $\,{B}_{x}\approx 155\,{\rm{mT}}$ (and similarly for negative fields of approximately the same magnitude), a drastic change of this regime is observed, with repeated field-induced transitions between a ${\rm{\phi }}=0$ SQUID and a ${\rm{\phi }}={\rm{\pi }}$ SQUID, as emphasized by the black arrows. Similar transitions are also observed in other weak links with strong spin-orbit coupling materials^19,20,46^.

**Figure 4:**
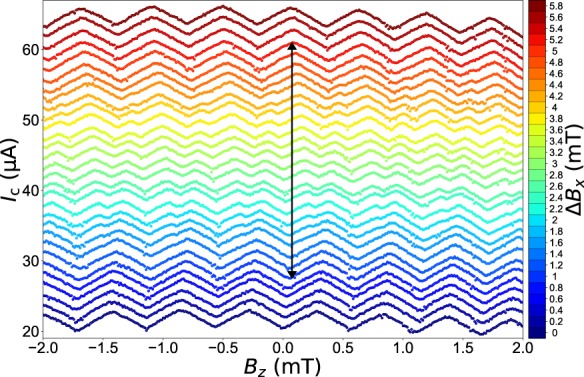
0-π-transition induced by the in-plane field. *I*_c_(*B*_*z*_) sweeps with additional in-plane field at the second 0-π-transition at ${B}_{x}={B}_{x0}+{\Delta }{B}_{x}\,\approx 220\,{\rm{mT}}$. The curves are vertically offset for clarity. The in-plane field induces a continuous 0-π-transition in the CPR, as indicated by the black arrow. The transition comes along with fractional flux states and, in particular, a half-integer periodicity is pointed out (green contours).

The transition at ${B}_{x}\approx 220\,{\rm{mT}}$ is shown in Fig. 4. Notably, the switching of the phase does not occur abruptly in *B*_*x*_, but takes shape in a finite range of ≈ 4 mT. The transition thus comes along with fractional flux periodicity. Particularly, the occurrence of a distinct half integer flux quantum state is stressed (within the range of green contours), which serves as a fingerprint of the boundary of a 0-π-transition. Such an effect has been theoretically predicted in closely related systems of Rashba-type spin-orbit coupled superconductors^37,47^.

**Figure 5:**
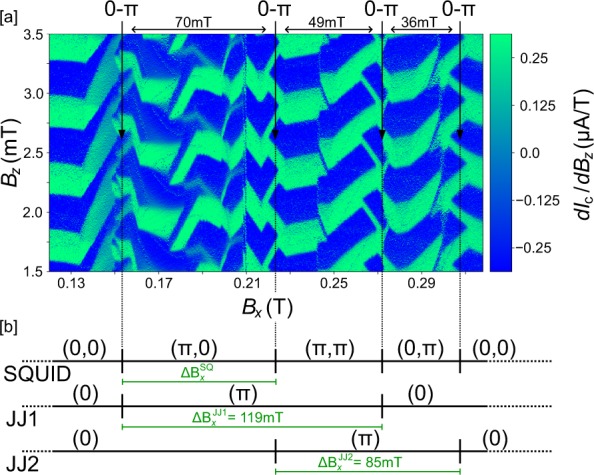
Transitional pattern in the in-plane field. (**a**) $d{I}_{{\rm{c}}}/d{B}_{z}({B}_{x},{B}_{z})\,$ data of the first four 0-π-transitions at *T* = 500 mK, including the one presented in Fig. 4. Transitions are observed at ${B}_{x}\approx \{153\,{\rm{mT}},\,220\,{\rm{mT}},\,272\,{\rm{mT}},$ $308\,{\rm{mT}}\}$. (**b**) Schematic illustration of the switching behaviour. Due to crystallographic and patterning-induced asymmetries, the two Josephson junctions are slightly asymmetric and undergo transitions at different onset fields and field modulation. This creates a regular pattern $(0,0)\to ({\rm{\pi }},0)\to ({\rm{\pi }},{\rm{\pi }})\to (0,{\rm{\pi }})\to (0,0)$ of the SQUID.

A closer look at the evolution of the transitional regime reveals the repeated occurrence of 0-π-transitions with similar spacing in field ${\Delta }{B}_{x}^{{\rm{SQ}}}$ for the first 4 transitions, as shown in Fig. 5a. We attribute this behavior to distinct 0-π-switches of the two weak links, which are patterned nominally symmetrical and should hence obey the same physics. However, they exhibit slightly different onset fields and spacing ${\Delta }{B}_{x}^{{\rm{JJ}}}$ due to microscopic patterning-induced and growth-related asymmetry. When one junction switches to a π-regime, the overall SQUID exhibits a π-shift, and when the second junction switches to a π-regime, the SQUID recovers a 0 state. This gives rise to the following transition pattern $(0,0)\to (0,{\rm{\pi }})\to ({\rm{\pi }},{\rm{\pi }})\to ({\rm{\pi }},0)\to (0,0)$ for the two junctions, as shown schematically in Fig. 5b. For larger values, the *B*_*x*_ superconductivity starts to be strongly suppressed as the critical field is approached.

As discussed by Hart *et al*.^19^, structural inversion symmetry (SIA), bulk inversion symmetry (BIA) and Zeeman effect coupling (ZEC), possibly modified by random phase distribution, can all lead to a spatially varying order parameter,. We should point out that there is pronounced phase drift in some regions between two complete 0-π transitions; for instance at ${B}_{x}\approx \{153\,{\rm{mT}},\,220\,{\rm{mT}},\,272\,{\rm{mT}},\,308\,{\rm{mT}}\}$. Random phase distribution might be responsible for a skewed Fraunhofer pattern and this is a viable explanation for the observed regions of phase drift in our SnTe-SQUIDs^19^.

While SIA and BIA lead to order parameter oscillations along the junction and perpendicular to the junction respectively, ZEC causes isotropic in-plane shift of the Cooper pair momentum^19^. Therefore, we argue that the behaviour of our system is dominated by the Zeeman coupling as our observed effect is similar in different in-plane field directions (see Supplementary Information). Nevertheless, *I*_c_(*B*_*y*_, *B*_*z*_) is not entirely the same as *I*_c_(*B*_*x*_, *B*_*z*_) and hence we assume that there is a SIA contribution which is responsible for this small mismatch.

It has been shown that DC SQUIDs with two purposefully asymmetric weak links with strong spin-orbit interaction exhibit 0-π-transitions as a function of the applied in-plane field in HgTe/HgCdTe^19,41^, Bi nanowire-based devices^20^ or BiSb topological semimetal^48^.

According to Hart *et al*.^19^, the onset magnetic field for a 0-π-transition in a single junction dominated by Zeeman coupling is ${B}_{{\rm{on}}}^{{\rm{JJ}}}=\frac{3\pi }{2}\frac{{\rm{\hslash }}{v}_{{\rm{F}}}}{g{\mu }_{{\rm{B}}}L}$. We use the expression $\frac{{\rm{\hslash }}{v}_{{\rm{F}}}}{L}={E}_{{\rm{th}}}$, where we substitute the Thouless energy extracted from the fit in Fig. 2a. This gives an estimate for the g-factor of SnTe of *g* ≈ 24 − 35 for the two junctions, respectively. The second 0-π-transition is predicted^19^ to be at ${B}_{2}^{{\rm{JJ}}}=\frac{5\pi }{2}\frac{{\rm{\hslash }}{v}_{{\rm{F}}}}{g{\mu }_{{\rm{B}}}L}$, which gives rise to a consistent *g* ≈ 29 − 33. The extracted g-factors are in line with SnTe literature values from simulations (g = 19–67)^49,50^ and experiments (g ≈ 57)^51^. Similarly high g-factor values are also reported for Bi nanowires^20^ and InSb^25^ nanowires showing 0-π-transitions.

## Conclusion

We have demonstrated assisted, reproducible 0-π transitions in SnTe-based SQUIDs in this article. The observed four transitions correlate well with theoretical prediction for induced Cooper pair momentum due to Zeeman coupling in the strong spin-orbit coupling material SnTe. The experimentally determined onset fields and field spacings between the 0-π transitions show remarkably close scaling agreement with the theoretical predictions and we have extracted *g* ≈ 30 for our SnTe weak links.

We believe that the observation of this unconventional effect will further fuel the interest in the integration of SnTe topological crystalline insulator in superconducting devices with new functionalities. Such field-tunable devices can be crucial components in proposed future topological quantum computing schemes.

## Methods

SnTe films of 40 nm thickness were grown by co-sputtering of Sn and Te on Si/SiO_2_ substrates in similar fashion to the previously presented work^24^ and the respective Supplementary Information. Here we use 30 nm films of chemically more stable Ta superconductor to proximity-induce Cooper pairing in the SnTe. Patterning of the samples is done with a positive-resist lift-off process for the Ta rings and a subsequent negative-resist argon milling process controlled by secondary ion mass spectroscopy to remove the excess SnTe and hence reduce the width of the weak links and stray current contributions.

## Supplementary information

Supplementary Information Field-Tunable 0-π-transitions in SnTe topological crystalline insulator SQUIDs

## Data Availability

The datasets generated during and/or analysed during the current study are available from the corresponding author on reasonable request.
