# Supplementary material for: Field-Tunable 0-π-Transitions in SnTe Topological Crystalline Insulator SQUIDs
Source: Sci Rep. 2019 Feb 13;9:1987. doi: 10.1038/s41598-018-38008-1 (PMC6374487; doi:10.1038/s41598-018-38008-1)
Supplement: Supplementary file 1 — Supplementary Information Field-Tunable 0-π-transitions in SnTe topological crystalline insulator SQUIDs [file 41598_2018_38008_MOESM1_ESM.docx]

## **Supplementary Information**

Field-Tunable 0-π-transitions in SnTe

topological crystalline insulator SQUIDs

Joachim Schönle^1,3^, Kiril Borisov^4^, Robin Klett^2^, Denis Dyck^2^, Franck Balestro^1^, Günter Reiss^2^, Wolfgang Wernsdorfer^1,3,4^

*^1^Institut Néel, CNRS and University Grenoble-Alpes, 25 Rue des Martyrs, F-38042 Grenoble, France*

*^2^Center for Spinelectronic Materials & Devices, Physics Department, Bielefeld University, Universitätsstraße 25, D-33615 Bielefeld, Germany*

*^3^Physikalisches Institut (PHI), Karlsruhe Institut of Technology (KIT), Wolfgang-Gaede-Straße 1, D-76131 Karlsruhe, Germany*

*^4^Institute of Nanotechnology (INT), Karlsruhe Institut of Technology (KIT), Hermann-von-Helmholtz-Platz 1, D-76334 Eggenstein-Leopoldshafen, Germany*

In this supporting material we further discuss the current-phase relation of the sample at different temperatures, the derivation of the normal resistance of the junctions as well as additional in-plane magnetic field measurements.

In the main text the occurrence of strongly reduced modulation depth and a triangular CPR $I_{c}(B_{z})$ was already discussed in the context of strong kinetic effects in the SnTe/Ta hybrid at the measurement temperature $T \cong500 \text{mK}$, presented again in fig.1c, where the kinetic inductance is given by $L_{\text{k}} \approx450 \text{pH}$ and the corresponding screening factor $\beta_{\text{k}}=\frac{{{2\pi L}_{\text{k}}I}_{\text{c}}}{\Phi_{0}} \approx20\gg\beta_{\text{geom}}$. Consistently, this kinetic screening increases towards lower temperatures as a result of increasing $I_{c}$, as shown for $T = 400 \text{mK}$ and $T=30 \text{mK}$ in fig.1b and fig.1a, respectively. Here, the kinetic effects give rise to multivalued switching.

For temperatures approaching critical temperature of the SnTe weak links $T_{\text{c}}^{\text{SnTe}} \approx900 \text{mK}$ the critical current and hence $\beta_{\text{k}}$ decreases. Furthermore, the weak link undergoes a transition^1^ from long junction characteristics at low temperatures to shorter junction characteristics closer to $T_{\text{c}}^{\text{SnTe}}$, as $L/\xi_{N}$ decreases with increasing $\xi_{N}$. As a result, the CPR shows the classical cosine-like flux dependence of the SQUID.

We can also extract the normal resistance of the weak links in this temperature regime close to the SnTe transition. Here, the critical current is strongly decreased and the switching does not affect the bulk superconducting structures via thermal avalanche. A similar effect is achieved by additional magnetic fields. In fig.2 the response to perpendicular magnetic fields is shown in a $R_{\mathrm{diff}}(B_{z},I)$ plot for $T = 770 \text{mK}$ and elevated fields $B_{z}$, representing the SQUID oscillation pattern with switching of the weak link structures only. From the first resistance plateau we can deduce $R_{\text{n}} \approx5 \Omega$ of the weak links, which is confirmed by supporting resistivity measurements of a SnTe Hall bar structure. At $I \approx4 \mu\text{A}$ a second transition is visible, which corresponds to the bulk Ta structures. This transition is only modulated very slowly in $B_{z}$.

In fig.3 we present in-plane magnetic field measurements at different angles to the ones along $B_{x}$ in the main text, namely along $B_{y}$ and at 45° in the $B_{x}$-$B_{y}$-plane. Similar to the case of $B_{x}$, a linear drift is corrected in the data. Again, 0-π-transitions are observed at onset fields of comparable magnitude, here at $B_{y} = 136 \mathrm{mT}$ and $B_{ip 45^{\circ}} = 169 \mathrm{mT}$, which supports a picture of isotropic Zeeman-induced spatially varying order parameter.^2^ Quantitative differences could be explained by geometrical reasons, random phase effects or contributions of SIA related to the microscopic crystallographic properties of the weak links. These values for the onset fields give rise to $g\approx40$ and $g\approx32$, respectively.

Further transitions are found for $B_{y} \approx\{230 \text{mT, 306}\text{ }\text{mT}\text{\}}$ and $B_{ip 45^{\circ}} \approx219 \text{mT}$, which find similar values as counterparts in the set of $B_{x}$-transitions. As it was already the case for $B_{x}$-in-plane fields, transitional features which do not lead to an overall phase change of π of the SQUID are observed as well, possibly as a result of field-modulated random phase effects and mitigating the occurrence of complete 0-π-transitions at certain field values which can hence not be identified with certainty.

## *References*

(1) Galletti, L. Coherence Effects in Superconducting Hybrid Devices. **2014**.

(2) Hart, S.; Ren, H.; Kosowsky, M.; Ben-Shach, G.; Leubner, P.; Brüne, C.; Buhmann, H.; Molenkamp, L. W.; Halperin, B. I.; Yacoby, A. Controlled Finite Momentum Pairing and Spatially Varying Order Parameter in Proximitized HgTe Quantum Wells. *Nat. Phys.* **2016**, *13* (1), 87–93.

*Figures*

**Fig.1)** Evolution of the CPR with temperature for 4 different values. At $T = 30 \text{mK}$ and, to a smaller extent, at $T = 400 \text{mK}$ multi-valued switching and strongly decreased modulation depth are observed as a result of large kinetic screening factors $\beta_{\text{k}}$. At $T = 675 \text{mK}$ the CPR is single-valued and triangular. Upon approaching $T_{\text{c}}^{\text{SnTe}}$ a further decrease of $\beta_{\text{k}}$ and a crossover to shorter junction characteristics restore the common cosine-like CPR.

**Fig.2)** Response to perpendicular magnetic fields $R_{\mathrm{diff}}(B_{z},I)$ measured at $T = 770 \text{mK}$. Clear SQUID oscillations are visible, with the first transition into a resistive state corresponding to the weak links. $R_{\text{n}} \approx5 \Omega$ of the weak links is extracted from this first resistance plateau. A second transition at $I \approx4 \mu\text{A}$ is observed, which is insensitive to $B_{z}$ (on small scales) and is assigned to the bulk Ta structures.

**Fig.3)** $I_{\text{c}}(B_{y},B_{z})$ (top panel) and $I_{\text{c}}(B_{ip 45^{\circ}},B_{z})$ measurement (bottom panel) showing the evolution of the SQUID modulation with different in-plane field angles. Again, the data is taken at $T = 500 \text{mK}$ and the linear drift corrected by rotation. The onset of the transitional regimes is found at $B_{y} = 136 \mathrm{mT}$ and $B_{ip 45^{\circ}} = 169 \mathrm{mT}$ with field-induced 0-π-transitions pointed out by the black arrows. Further features are observed but are not identified as full transitions in these data sets.
